# The Nonlinear Relationship Between Temperature and Prognosis in Sepsis-induced Coagulopathy Patients: A Retrospective Cohort Study from MIMIC-IV Database

**DOI:** 10.5811/westjem.18589

**Published:** 2024-08-16

**Authors:** Guojun Chen, Tianen Zhou, Jingtao Xu, Qiaohua Hu, Jun Jiang, Weigan Xu

**Affiliations:** * First People’s Hospital of Foshan, Department of Emergency, Foshan, Guangdong, China; † First People’s Hospital of Foshan, The Poison Treatment Centre of Foshan, Foshan, Guangdong, China; ∘ Guojun Chen and Tianen Zhou are co-first authors and contributed equally to this work

## Abstract

**Background:**

The prognostic value of body temperature in sepsis-induced coagulopathy (SIC) remains unclear. In this study we aimed to investigate the association between temperature and mortality among SIC patients.

**Methods:**

We analyzed data for 9,860 SIC patients from an intensive care database. Patients were categorized by maximum temperature in the first 24 hours into the following: ≤36.0°C; 36.0–37.0°C; 37.0–38.0°C; 38.0–39.0°C; and ≥39.0°C. The primary outcome was 28-day mortality. We used multivariate regression to analyze the temperature-mortality association.

**Results:**

The 37.0–38.0°C, 38.0–39.0°C and ≥39.0°C groups correlated with lower 28-day mortality (adjusted HR 0.70, 0.76 and 0.72, respectively), while the <36.0°C group correlated with higher mortality compared to the 36.0–37.0°C group (adjusted HR 2.60). A nonlinear relationship was observed between temperature and mortality. Subgroup analysis found no effect modification except in cerebrovascular disease.

**Conclusion:**

A body temperature in the range of 37.0–38.0°C was associated with a significantly lower mortality compared to the normal temperature (36.0–37.0°C) group. Additionally, a gradual but statistically insignificant increase in mortality risk was observed when body temperature exceeded 38.0°C. Further research should validate these findings and elucidate involved mechanisms, especially in cerebrovascular disease subgroups.

Population Health Research Capsule What do we already know about this issue?

*Body temperature is a critical vital sign, but its prognostic value in sepsis-induced coagulopathy (SIC) remains unclear, with conflicting evidence on ideal temperature ranges.*

What was the research question?

*We aimed to investigate the association between body temperature and 28-day mortality among SIC patients.*

What was the major finding of the study?

*Compared to 36.0–37.0°C, 37.0–38.0°C was associated with lower mortality (adjusted HR 0.70, 95% CI 0.62–0.79, P < 0.001).*

How does this improve population health?

*Identifying the optimal temperature range in SIC could guide better thermoregulation management, potentially reducing mortality and improving outcomes in this high-risk population.*

## INTRODUCTION

Sepsis, defined as life-threatening organ dysfunction due to dysregulated host response to infection,^1^ frequently leads to derangements in coagulation ranging from subtle activation to overt, disseminated intravascular coagulation (DIC).^2^ ^,^ ^3^ This condition in sepsis is associated with multiple organ failure and high mortality.^4^ ^,^ ^5^ In 2017 the International Society on Thrombosis and Haemostasis (ISTH) proposed “sepsis-induced coagulopathy” (SIC) criteria to identify early coagulopathy in sepsis, defined by sepsis plus thrombocytopenia and prolonged prothrombin time.^4^ Several studies have validated SIC as an early identifier of impending overt DIC in sepsis.^6^ ^,^ ^7^ Sepsis-induced coagulopathy correlates with mortality, with rates exceeding 30% at a score ≥4.^4^ Compared to overt DIC criteria, SIC demonstrates greater sensitivity in predicting mortality.^7^

In summary, SIC represents an early phase of coagulation dysfunction in sepsis that often progresses to overt DIC. The high morbidity and mortality associated with SIC highlights the need for early identification using simple criteria such as the SIC score, allowing rapid initiation of interventions that may improve outcomes in this high-risk population. Monitoring body temperature changes is vital in evaluating septic patient prognosis, as fever is a common symptom.^8^ Both hypothermia and hyperthermia in sepsis reflect immune response to infection. However, their implications for prognosis remain debated.^9^ ^–^ ^13^

Previous studies have associated hypothermia with poor outcomes.^14^ ^–^ ^19^ The prognostic value of hyperthermia is less clear, with conflicting reports.^11^ ^,^ ^13^ ^,^ ^20^ This highlights the need to clarify the role of body temperature in sepsis.

Considering that SIC significantly influences sepsis prognosis by altering coagulation,^4^ ^,^ ^21^ ^,^ ^22^ an exploration of the relationship between temperature and SIC will enhance our comprehension of sepsis pathophysiology and facilitate more precise prognostication and management strategies. Nonetheless, the prognostic value of body temperature abnormalities specifically within SIC has not been comprehensively established. Therefore, we aimed to investigate the association between body temperature and the short term mortality of septic patients with coagulopathy. The findings are expected to bridge a critical knowledge gap concerning the intricate interplay between temperature regulation and coagulation in sepsis.

## MATERIALS AND METHODS

### Data Source

The data utilized in this study was extracted from the Medical Information Mart for Intensive Care-IV (MIMIC-IV) database through the employment of Navicat Premium 15 software. MIMIC-IV, a collaborative effort between Beth Israel Deaconess Medical Center (BIDMC) and Massachusetts Institute of Technology (MIT), is a comprehensive repository encompassing data from over 60,000 adult intensive care unit (ICU) admissions at Beth Israel Deaconess Medical Center spanning from 2008 to 2019.^23^ The data, collected during routine clinical care at BIDMC, were de-identified, transformed, and made accessible to researchers who have completed requisite training in human research ethics and entered into a data use agreement. Access to the database was secured by our corresponding author, bearing certification No. 46450588. The Institutional Review Board at BIDMC granted an exemption from obtaining informed consent and approved the sharing of the research resource.

### Study Population

Patient data conforming to sepsis-induced coagulopathy criteria were extracted from the MIMIC-IV database’s ICU records. The patient selection process for this study adhered to the following inclusion criteria: (1) hospital stay duration of ≥24 hours; (2) age ≥18 years. Exclusion criteria encompassed: (1) presence of greater than 10% missing individual data; (2) outliers, indicated by values exceeding the mean ± 3 Standard Deviation (SD). In cases of multiple ICU admissions, solely data from the first ICU admission of the initial hospital stay were considered.

### Data Extraction

Structured Query Language (SQL) was harnessed to extract data recorded on the first day of admission. Extracted data included demographic details, fundamental vital signs, comorbidities, basic laboratory parameters, and pre-treatment scoring systems. Demographic data encompassed race, gender, and age. Vital signs incorporated heart rate, respiratory rate, and body temperature. Body temperature was routinely measured by nurses at bedside, primarily through the axillary and oral route, at 4–8 hour intervals. For some ICU patients, occasional core temperatures from esophageal and rectal probes were also available. We extracted the maximum temperature on day one for each patient. Comorbidities were categorized as myocardial infarction, congestive heart failure, cerebrovascular disease, chronic pulmonary disease, renal disease, and diabetes. Laboratory parameters encompassed hemoglobin, platelet count, white blood cell count, bicarbonate, creatinine, urea nitrogen, glucose, Prothrombin Time (PT), Partial Thromboplastin Time (PTT), and International Normalized Ratio (INR). Additionally, the Charlson Comorbidity Index (CMI), Sequential Organ Failure Assessment (SOFA) score, Simplified Acute Physiology Score II (SAPSII), Renal Replacement Therapy (RRT), first-day ventilation use, and vasopressor use were included. Variables with missing values ≥40% were excluded during variable selection. For some of the continuous variables included with missing data (see the Supplementary Table S1 for missing data), we use the simple substitution method to deal with them. Missing values are replaced with means if they follow a normal or approximately normal distribution, and with medians if they follow a skewed distribution.

### Primary and Secondary Outcomes

The primary outcome measure was 28-day mortality, while secondary outcomes included 90-day mortality, length of stay (LOS) in both the Intensive Care Unit (ICU), and hospital.

### Statistical Analysis

All participants were categorized into five groups based on their maximum body temperature within the first 24 hours of hospital admission: ≤36.0°C, 36.0–37.0°C, 37.0–38.0°C, 38.0–39.0°C, and ≥39.0°C. The reference group was defined as the temperature category of 36.0–37.0°C. Normally distributed continuous variables were presented as mean ± standard deviation, while non-normally distributed data were displayed as median [interquartile range (IQR)]. Normality distribution was assessed using the Shapiro-Wilk test. Categorical data were presented as counts (percentages). To compare the differences across groups, one-way analyses of variance (normal distribution), Kruskal–Wallis tests (skewed distribution), and chi-square tests (categorical variables) were undertaken.

Univariate logistic regression analyses were performed to explore the associations between each variable and the risks of 28-day and 90-day mortality, with the results presented in Supplementary Tables S2 and S3, respectively.

The association between body temperature and 28-day mortality was examined via Cox proportional hazard regression, with results expressed as Hazard Ratios (HR) along with 95% Confidence Intervals (CI). For each endpoint, four multivariate analytic models were developed: Model 1, unadjusted covariates; Model 2, covariates including gender, age, race, heart rate, and respiratory rate; Model 3, further adjusted for hemoglobin, platelet count, INR, white blood cell count, anion gap, bicarbonate, blood urea nitrogen, creatinine, glucose, PT, and PTT; and Model 4, encompassing all covariates. Linear trend tests across temperature categories were performed using the median temperature value in each group.

We also examined the linearity of the body temperature-mortality association using curve fitting. For this analysis, we modeled temperature as a continuous variable, rather than using the pre-defined temperature categories. A two-piecewise Cox model with smoothing spline was used to determine the threshold relationship between body temperature and mortality, with the threshold point identified through likelihood ratio testing and bootstrap resampling.

Furthermore, potential modifications of the relationship between body temperature and mortality were assessed, including the following variables: age (<70 vs. ≥70 years), gender, SOFA score (<4 vs. ≥4), SAPSII (<42 vs. ≥42), myocardial infarct (yes vs. no), cerebrovascular disease (yes vs. no), chronic pulmonary disease (yes vs. no), diabetes mellitus (yes vs. no) and renal disease (yes vs. no). Heterogeneity among subgroups was assessed by multivariate cox regression, and interactions between subgroups and body temperature were examined by likelihood ratio testing. To adjust for multiple comparisons in these subgroup analyses, we applied the Bonferroni correction. Specifically, we divided the predetermined significance level of α = 0.05 by the number of subgroup comparisons (9), resulting in a Bonferroni-adjusted significance level of 0.0056. We then evaluated whether the association between temperature and SIC mortality differed significantly in each subgroup, using this corrected significance threshold.

Finally, Kaplan-Meier curves were constructed to visually examine the association between temperature categories and mortality.

Analyses were conducted using R software (http://www.R-project.org, The R Foundation) and Free Statistics software (version1.7), with two-sided *p* < 0.05 considered statistically significant.

We used Claude AI, an artificial intelligence writing assistant developed by Anthropic, to aid in final polishing of the manuscript. Claude AI provided suggestive content which we then reviewed, edited, and approved before inclusion. We take full responsibility for the content and conclusions of the manuscript.

## RESULT

### Baseline Characteristics and Patient Outcome

**Figure 1.**
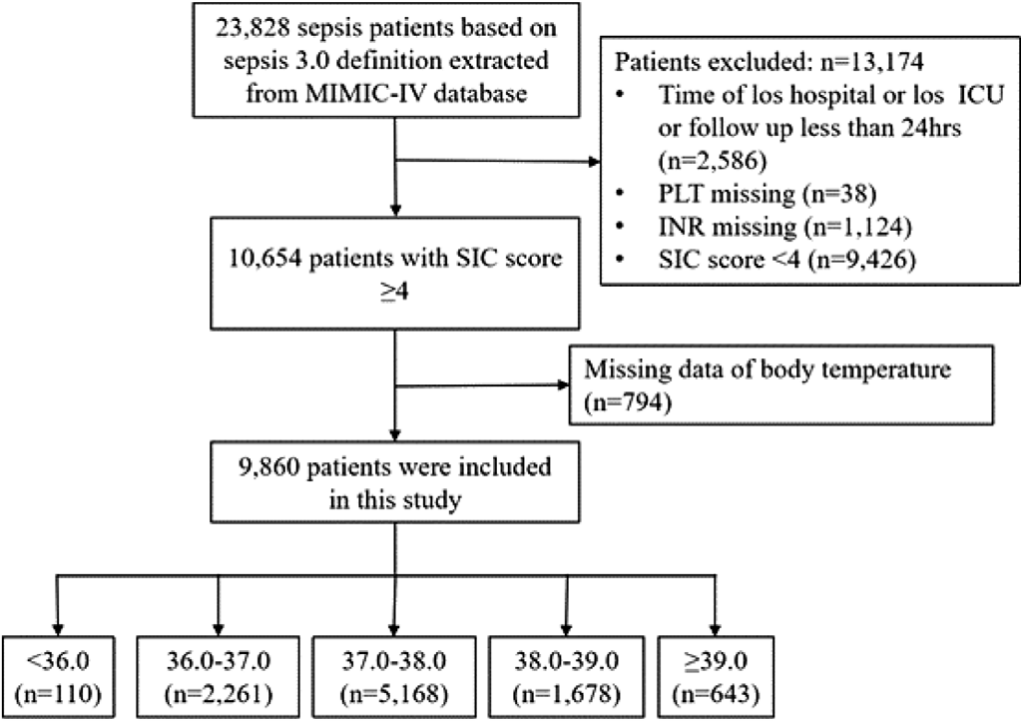
Flowchart of the screening and enrollment of study participants. MIMIC: Medical Information Mart for Intensive Care. *ICU*, Intensive Care Unit; *LOS*, length of stay; *PLT*, platelet; *INR*, International Normalized Ratio; *SIC*, sepsis-induced coagulopathy.

A total of 9,860 patients diagnosed with sepsis-induced coagulopathy (SIC) were included in this comprehensive study (Figure 1). The demographic details, vital signs, laboratory parameters, and comorbidities of these patients at baseline are meticulously presented in Table 1. Notably, compared to patients with a body temperature between 38.0–39.0°C, the mortality rate was slightly higher in the group with body temperatures above 39°C. However, the highest mortality rate was observed in the group with body temperatures below 36.0°C, suggesting a bimodal relationship between body temperature and mortality in SIC patients.

**Table 1.** Baseline clinical and laboratory characteristics of the study patients.

	Temperature (°C)						
	Total	<36.0	36.0 ∼ < 37.0	37.0 ∼ < 38.0	38.0 ∼ < 39.0	≥39.0	
Characteristic	n = 9,860	n = 110	n = 2,261	n = 5,168	n = 1,678	n = 643	*P*-value
Age, mean ± SD	66.9 ± 15.9	65.3 ± 18.2	70.7 ± 14.7	67.5 ± 15.2	63.5 ± 16.8	57.7 ± 17.9	< 0.001
Race, n (%)							< 0.001
White	6,730 (68.3)	62 (56.4)	1,605 (71)	3,546 (68.6)	1,149 (68.5)	368 (57.2)	
Other	3,130 (31.7)	48 (43.6)	656 (29)	1,622 (31.4)	529 (31.5)	275 (42.8)	
Gender, n (%)							< 0.001
Male	6,049 (61.3)	66 (60)	1,291 (57.1)	3,192 (61.8)	1,089 (64.9)	411 (63.9)	
Female	3,811 (38.7)	44 (40)	970 (42.9)	1,976 (38.2)	589 (35.1)	232 (36.1)	
Vital sign							
HR, mean ± SD	107.4 ± 21.6	99.0 ± 19.1	102.6 ± 21.2	105.4 ± 20.4	114.2 ± 20.7	124.4 ± 22.9	< 0.001
RR, mean ± SD	28.7 ± 6.7	27.3 ± 5.4	27.6 ± 5.9	28.4 ± 6.6	29.8 ± 6.7	32.8 ± 7.6	< 0.001
Laboratory data							
WBC (×10^9^/L), mean ± SD	16.0 ± 14.5	18.0 ± 8.9	15.2 ± 10.6	16.0 ± 13.9	16.3 ± 12.8	18.4 ± 29.2	< 0.001
HGB (g/dL), median (IQR)	9.2 (7.9, 10.7)	9.2 (8.2, 11.5)	9.1 (7.8, 10.5)	9.1 (7.9, 10.6)	9.4 (8.0, 10.9)	9.6 (8.0, 11.1)	< 0.001
AG (mEq/L), mean ± SD	12.9 ± 3.8	15.2 ± 4.6	13.4 ± 4.1	12.8 ± 3.8	12.6 ± 3.6	13.1 ± 3.6	< 0.001
CO_2_ (mmol/L), mean ± SD	20.4 ± 4.8	16.7 ± 5.3	20.4 ± 5.0	20.6 ± 4.7	20.6 ± 4.6	19.4 ± 4.8	< 0.001
PLT (×10^9^/L), median (IQR)	117.0 (80.0, 164.0)	133.0 (100.0, 181.0)	118.0 (81.0, 175.0)	115.0 (80.0, 159.0)	118.0 (83.0, 165.0)	116.0 (67.0, 164.0)	0.001
Glucose (mg/dL), median (IQR)	143.0 (117.0, 190.0)	203.0 (141.0, 323.8)	142.0 (115.0, 188.0)	141.0 (116.0, 187.0)	147.5 (120.0, 194.0)	154.0 (122.0, 199.0)	< 0.001
BUN (mg/dL), median (IQR)	24.0 (16.0, 41.0)	29.5 (19.2, 55.2)	29.0 (18.0, 51.0)	24.0 (16.0, 39.0)	22.0 (15.0, 35.0)	22.0 (14.5, 37.0)	< 0.001
Scr (mg/dL), median (IQR)	1.2 (0.9, 2.0)	1.6 (1.1, 3.4)	1.3 (0.9, 2.2)	1.2 (0.8, 1.8)	1.2 (0.9, 1.8)	1.2 (0.9, 2.0)	< 0.001
INR, median (IQR)	1.6 (1.4, 2.0)	1.7 (1.5, 2.6)	1.7 (1.4, 2.3)	1.6 (1.4, 2.0)	1.6 (1.4, 2.0)	1.6 (1.4, 1.9)	< 0.001
PT (s), median (IQR)	17.5 (15.5, 22.1)	18.9 (16.1, 27.6)	18.1 (15.8, 24.3)	17.3 (15.4, 21.7)	17.2 (15.3, 21.1)	17.5 (15.6, 20.9)	< 0.001
PTT (s), median (IQR)	36.7 (31.1, 49.9)	47.8 (36.8, 85.4)	38.7 (32.1, 54.5)	36.2 (30.9, 48.8)	36.0 (30.9, 47.4)	35.9 (30.8, 48.0)	< 0.001
Medical history							
Myocardial infarct, n (%)	1,712 (17.4)	31 (28.2)	456 (20.2)	865 (16.7)	274 (16.3)	86 (13.4)	< 0.001
Congestive heart failure, n (%)	2,994 (30.4)	43 (39.1)	842 (37.2)	1,510 (29.2)	444 (26.5)	155 (24.1)	< 0.001
Cerebrovascular disease, n (%)	1,138 (11.5)	13 (11.8)	221 (9.8)	598 (11.6)	223 (13.3)	83 (12.9)	0.011
Chronic pulmonary disease, n (%)	2,379 (24.1)	30 (27.3)	578 (25.6)	1,269 (24.6)	373 (22.2)	129 (20.1)	0.012
Diabetes, n (%)	2,901 (29.4)	22 (20)	705 (31.2)	1,533 (29.7)	469 (27.9)	172 (26.7)	0.016
Renal disease, n (%)	2,161 (21.9)	31 (28.2)	647 (28.6)	1,093 (21.1)	297 (17.7)	93 (14.5)	< 0.001
Disease severity score							
Charlson comorbidity index, mean ± SD	6.0 ± 2.9	5.7 ± 2.9	6.6 ± 2.7	6.1 ± 2.8	5.4 ± 2.9	4.9 ± 3.0	< 0.001
SOFA score, mean ± SD	4.1 ± 2.3	4.8 ± 2.4	4.2 ± 2.3	4.1 ± 2.3	3.9 ± 2.1	4.1 ± 2.2	< 0.001
SAPSII, mean ± SD	41.7 ± 14.5	51.6 ± 16.6	43.2 ± 13.8	40.8 ± 14.2	40.7 ± 15.0	43.9 ± 15.9	< 0.001
Procedure							
RRT use, n (%)	605 (6.1)	19 (17.3)	157 (6.9)	292 (5.7)	88 (5.2)	49 (7.6)	< 0.001
Ventilator use, n (%)	5,065 (51.4)	87 (79.1)	879 (38.9)	2,667 (51.6)	1,063 (63.3)	369 (57.4)	< 0.001
Vasopressor use, n (%)	5,341 (54.2)	78 (70.9)	1,193 (52.8)	2,796 (54.1)	917 (54.6)	357 (55.5)	0.005
Outcomes							
Los hospital (days), median (IQR) days	9.0 (5.6, 16.0)	6.8 (3.9, 14.2)	8.5 (5.3, 15.1)	8.7 (5.4, 15.0)	10.0 (6.0, 18.2)	11.2 (6.8, 21.4)	< 0.001
Los ICU (days), median (IQR) days	3.1 (1.8, 6.2)	4.4 (2.6, 7.4)	2.9 (1.7, 5.3)	2.9 (1.7, 5.6)	3.8 (2.0, 7.7)	4.8 (2.5, 9.3)	< 0.001
28-day mortality, n (%)	2,062 (20.9)	61 (55.5)	622 (27.5)	938 (18.2)	305 (18.2)	136 (21.2)	< 0.001
90-day mortality, n (%)	2,754 (27.9)	68 (61.8)	811 (35.9)	1,292 (25)	408 (24.3)	175 (27.2)	< 0.001

*HR*, heart rate; *RR*, respiratory rate; *WBC*, white blood cell; *HGB*, hemoglobin; *PLT*, platelets; *AG*, anion gap; *CO_2_,* bicarbonate; *BUN*, blood urea nitrogen; *Scr*, serum creatinine; *INR*, International Normalized Ratio; *PT*, prothrombin time; *PTT*, partial thromboplastin time; *SOFA*, sequential organ failure assessment; *SAPSII,* simplified acute physiology score II; *RRT,* renal replacement therapy; *LOS,* length of stay.

### Relationships Between Body Temperature and Outcomes

Table 2 offers a comprehensive overview of the outcomes’ association with varying body temperatures. In the unadjusted model (Model 1), the hazard ratios (HRs) and 95% confidence intervals (CIs) for 28-day all-cause mortality were 2.82 (2.17, 3.66) for temperatures <36.0;°C, 0.62 (0.56, 0.69) for 37.0–38.0°C, 0.63 (0.55, 0.72) for 38.0–39.0°C, and 0.75 (0.62, 0.90) for ≥39.0°C, all compared to the reference group of 36.0–37.0°C.

**Table 2.** Results of multivariate regression analysis between temperature and outcomes.

	Model I		Model IV	
Variable	HR (95% CI)	*P*-value	HR (95% CI)	*P*-value
Primary outcomes				
28-day mortality^a^				
Temperature	0.78 (0.74 ∼ 0.83)	<0.001	0.82 (0.78 ∼ 0.87)	0.001
Body temperature				
<36.0	2.82 (2.17 ∼ 3.66)	<0.001	2.6 (1.99 ∼ 3.42)	<0.001
36.0–37.0	1 (Ref)		1 (Ref)	
37.0–38.0	0.62 (0.56 ∼ 0.69)	<0.001	0.7 (0.63 ∼ 0.78)	<0.001
38.0 ∼ −39.0	0.63 (0.55 ∼ 0.72)	<0.001	0.76 (0.66 ∼ 0.88)	0.001
≥39.0	0.75 (0.62 ∼ 0.9)	0.002	0.72 (0.59 ∼ 0.87)	0.001
Trend		<0.001		<0.001
Secondary outcomes				
90-day mortality				
Temperature	0.79 (0.75 ∼ 0.83)	<0.001	0.84 (0.8 ∼ 0.89)	<0.001
Body temperature				
<36.0	2.52 (1.97 ∼ 3.23)	<0.001	2.48 (1.92 ∼ 3.2)	<0.001
36.0–37.0	1 (Ref)		1 (Ref)	
37.0–38.0	0.64 (0.59 ∼ 0.7)	<0.001	0.73 (0.67 ∼ 0.8)	<0.001
38.0–39.0	0.63 (0.56 ∼ 0.71)	<0.001	0.79 (0.69 ∼ 0.89)	<0.001
≥39.0	0.72 (0.61 ∼ 0.85)	<0.001	0.73 (0.62 ∼ 0.87)	0.001
Trend		<0.001		<0.001
LOS ICU^b^	0.77^*^ (0.61 ∼ 0.92)	<0.001	0.4^*^ (0.24 ∼ 0.55)	<0.001
LOS hospital^b^	1.33^*^ (1.01 ∼ 1.66)	<0.001	0.82^*^ (0.48 ∼ 1.15)	<0.001

*Note:* Model I adjusted for nothing. Model IV adjusted for gender, age, race, HR, RR, hemoglobin, platelets, INR, WBC, anion gap, bicarbonate, bun, creatinine, glucose, PT, PTT, myocardial infarction, congestive heart failure, cerebrovascular disease, chronic pulmonary disease, renal disease, Charlson comorbidity index, SOFA score, SAPSII, RRT, first day ventilation use and vasopressor use.

a Logistic regression analysis.

b Linear regression analysis.

* Regression coefficient (β).

*HR*, hazard ratio; *CI*, confidence interval; *LOS*, length of stay.

In the fully adjusted Model 4, which accounted for gender, age, race, vital signs, laboratory parameters, comorbidities, and severity scores, the association between temperature and 28-day mortality remained statistically significant. This suggests a robust inverse relationship between body temperature and short-term mortality risk in this patient population.

The results from the additional regression models (Models 2 and 3) were provided in Supplementary Table S4. These models showed consistent findings, with higher temperatures being associated with lower mortality risk.

When temperature was examined as a continuous variable, the fully adjusted Model 4 showed that for every 1°C increase in body temperature, there was an 18% (HR 0.82, 95% CI 0.78–0.87) reduction in the risk of 28-day mortality.

**Figure 2.**
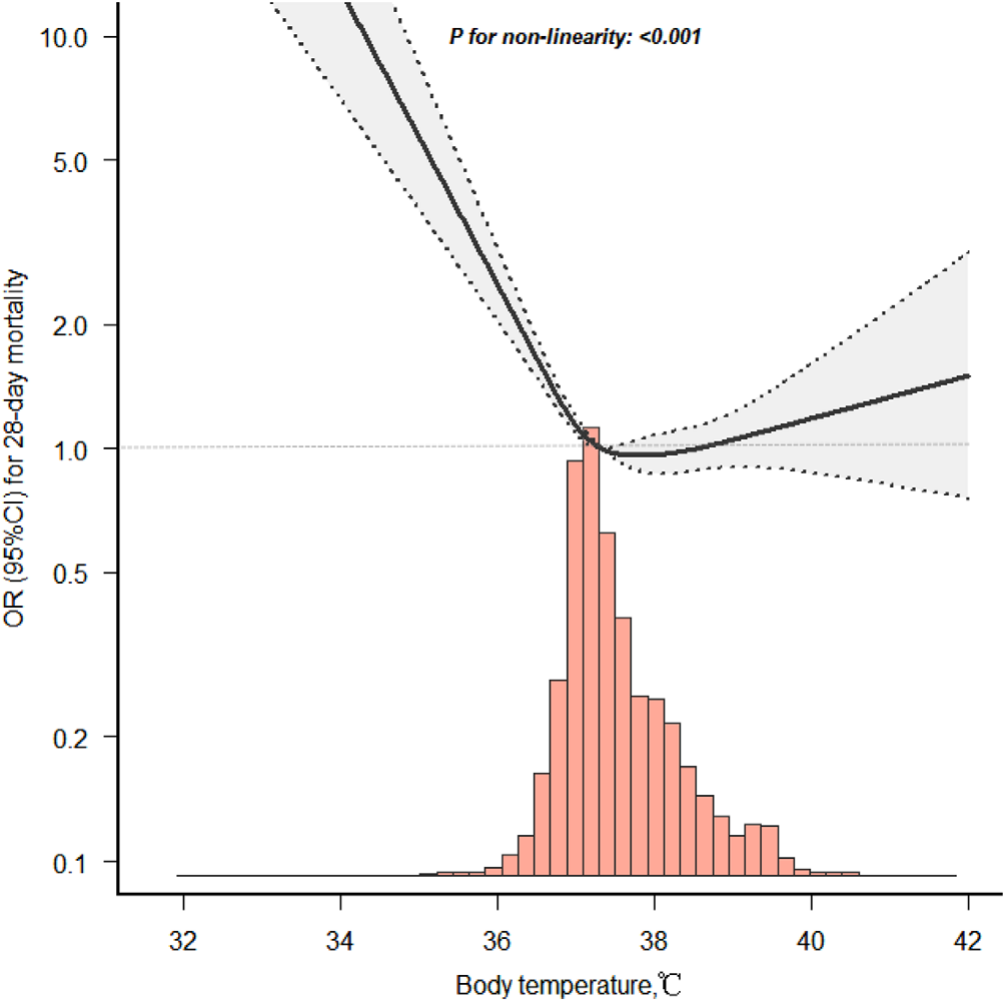
Smooth curve fitting for temperature and 28-day mortality in patients with sepsis-induced coagulopathy.

The non-linear relationship between temperature and 28-day all-cause mortality was further demonstrated by the smooth curve fitting analysis depicted in Figure 2 (*p* for non-linearity <0.001). A similar protective association was also observed for 90-day all-cause mortality (Supplementary Figure S1).

Furthermore, our multivariate linear regression models revealed that for every 1°C increase in body temperature, there was a corresponding increase in ICU length of stay by 0.40 days (95% CI: 0.24–0.55 days) and total hospital length of stay by 0.82 days (95% CI: 0.48–1.15 days) in the fully adjusted analysis.

### Threshold Effect Analysis of Body Temperature on SIC Patients’ Mortality

We conducted a smoothing function analysis to assess the potential non-linear relationship between body temperature and 28-day mortality in SIC patients. After adjusting for potential confounders including age, gender, laboratory results and comorbidities, we observed a non-linear association between body temperature and risk of 28-day mortality (Figure 2). This non-linear relationship was also observed when analyzing 90-day mortality (Supplementary Figure S1).

Further spline analysis revealed a threshold effect, with risk decreasing as body temperature increased up to 38.0°C (adjusted HR 0.640; 95% CI 0.589–0.696), after which risk slightly increased but was no longer statistically significant (adjusted HR 1.192; 95% CI 0.984–1.444). (Table 3)

**Table 3.** Threshold effect analysis of the association between the body temperature and the 28-day mortality in SIC patients.

Threshold of body temperature	HR (95%CI)
<38.095	0.640 (0.589, 0.696)
≥38.095	1.192 (0.984, 1.444)

*Note*: The data have been adjusted for all of the factors included in Model IV in Table 2.

*HR,* hazard ratio; *CI,* confidence interval; *SIC,* sepsis-induced coagulopathy.

The threshold effect at 38.0°C is illustrated by the spline curve in Figure 2, which depicts the estimated adjusted hazard ratios (solid line) and pointwise 95% confidence intervals (shaded area) for the relationship between body temperature and 28-day mortality risk. Our analyses indicate a complex non-linear association between body temperature and mortality risk in SIC patients, with hypothermia conferring the highest risk.

### Subgroup Analysis

**Figure 3.**
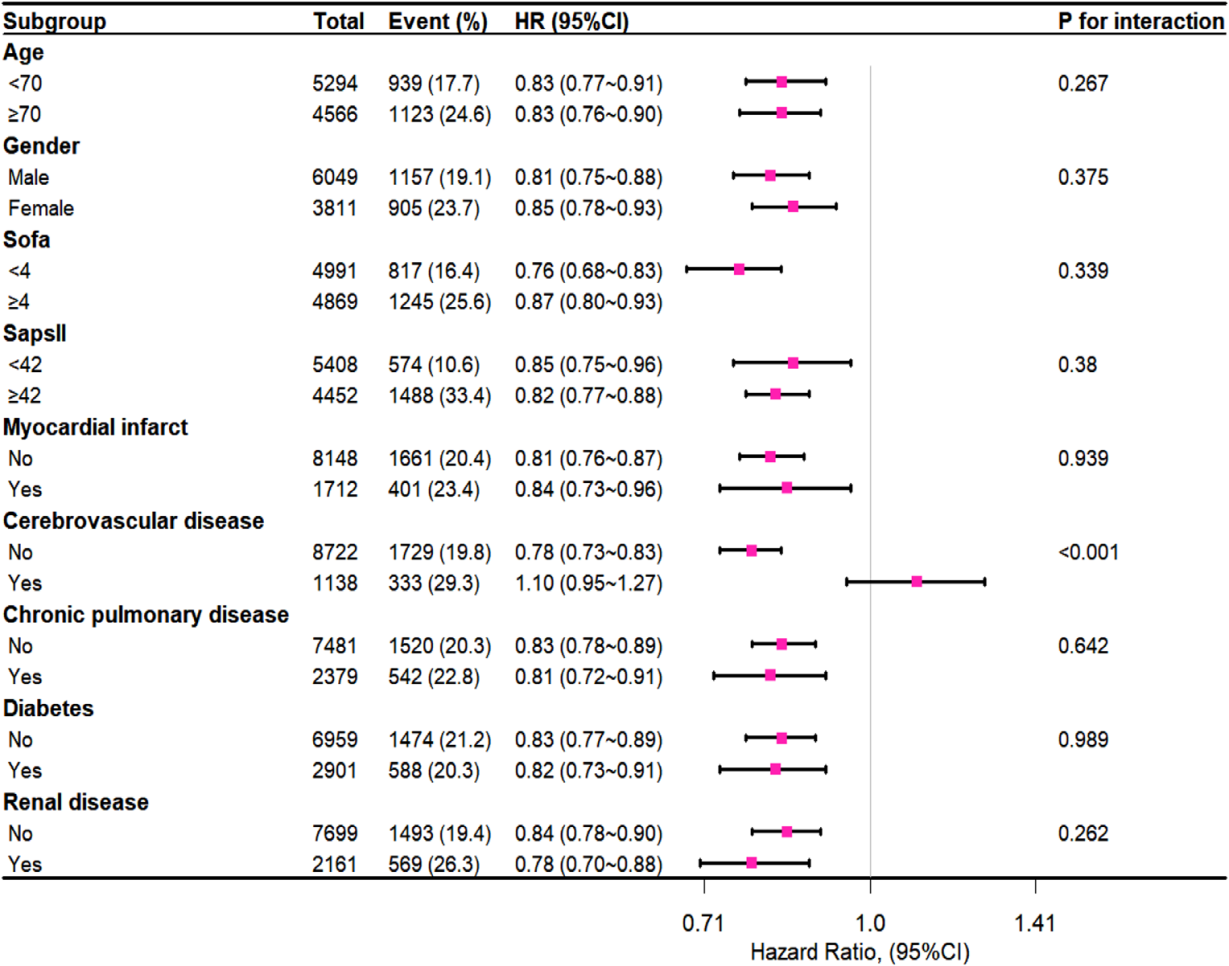
Subgroup analysis of association between temperature and 28-day mortality.

To identify factors impacting the effect of body temperature on 28-day mortality, comprehensive subgroup analyses were conducted, as depicted in Figure 3. Interaction *p*-values were largely non-significant across age, gender, severity scores, comorbidities, and other subgroups. Notably, an exception was observed for the cerebrovascular disease subgroup (p < 0.001), where patients without cerebrovascular disease exhibited a lower 28-day mortality risk (adjusted HR: 0.78, 95% CI 0.73∼0.83). Analogous findings are discernible in the 90-day subgroup analysis (Supplementary Figure S2).

### Survival Analysis

**Figure 4.**
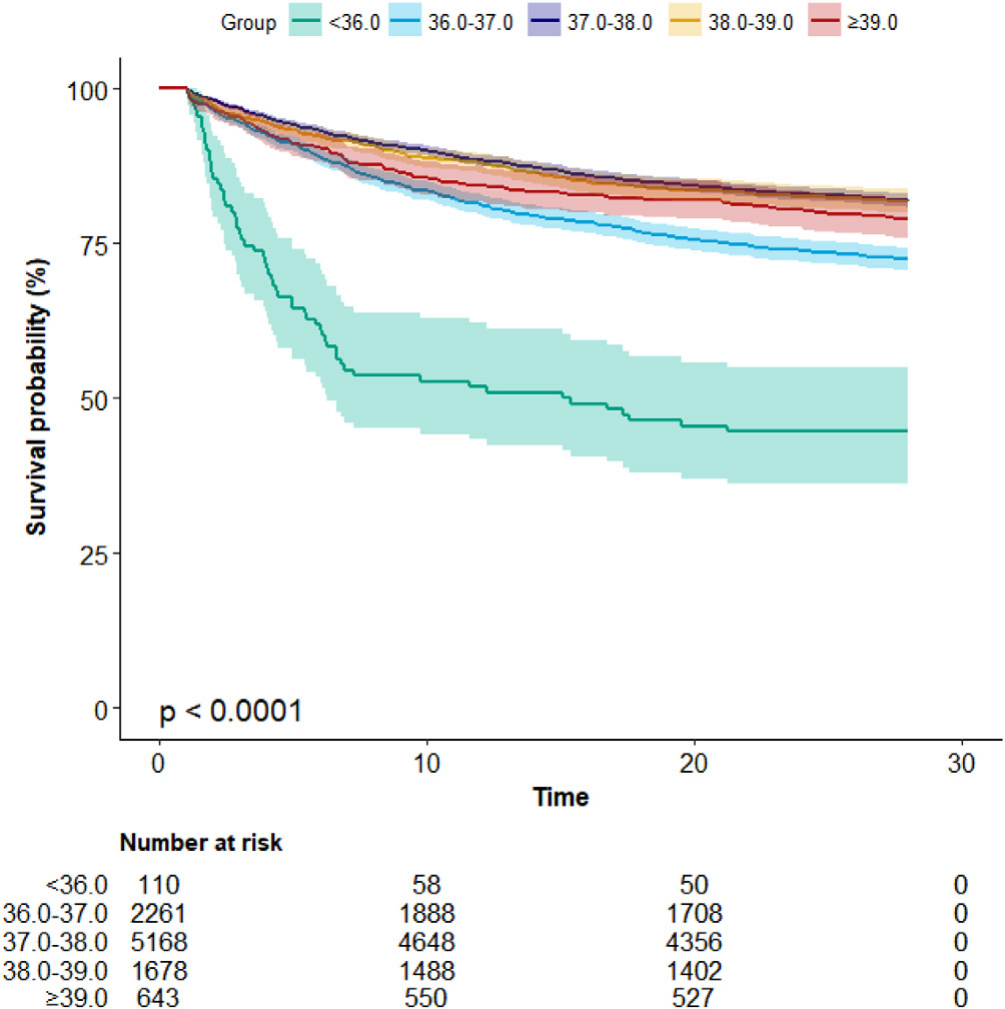
Kaplan–Meier curve of 28-day mortality for patients with sepsis-induced coagulopathy.

The Kaplan–Meier survival analysis in Figure 4 highlights the impact of varying body temperatures on 28-day survival. Mortality rates were notably higher in the <36.0 group and comparatively lower in the 37.0–38.0 group when contrasted with the reference group (36.0–37.0). This trend is reaffirmed with adjusted HRs of 2.6 (95% CI 1.99 ∼ 3.42) and 0.7 (95% CI 0.63 ∼ 0.78), respectively (Table 2). Consistent outcomes are observed in the 90-day survival analysis (Supplementary Figure S3).

## DISCUSSION

In our study, a significant revelation emerges from the analysis. Specifically, SIC patients within the 37.0–38°C temperature range exhibited the most favorable prognosis, characterized by the lowest mortality. Conversely, low body temperature (<36.0°C) was associated with higher mortality in the SIC population similar to that observed in other septic cohorts. Subgroup analyses, aside from cerebrovascular disease, demonstrated no substantial effect modification by age, gender, or comorbidities.

Our results contribute novel insights into the implications of body temperature in SIC, marking a pioneering attempt at investigating this association. Sepsis-induced coagulopathy holds a pivotal place in the spectrum of sepsis-related coagulation disorders, intimately connected with disseminated intravascular coagulation (DIC).^24^ Recognized for its role in the pathogenesis of sepsis, SIC acts as a bridge between early systemic inflammation and full-blown DIC. By highlighting the potential value of SIC as a marker and a possible therapeutic target, our study underscores its significance in guiding clinical decisions and furthering our understanding of sepsis pathogenesis.

The observed differences in mortality and hospital stay duration based on temperature groups underscore the clinical significance of temperature management in SIC. Patients in the hypothermic group (<36.0°C) displayed the highest 28-day mortality risk, suggestive of severe physiological distress and compromised organ function.^25^ Correspondingly, maintaining a normothermic range (37.0–38.0°C) was associated with improved survival outcomes, highlighting the potential importance of normothermia in enhancing patient prognosis.^26^ Our study echoes previous reports associating lower temperatures with heightened mortality risk in septic patients.^13^ ^,^ ^19^ Notably, our definition of hypothermia (<36.0°C) within the SIC cohort yielded a 2.6-fold increase in mortality after adjusting for confounding factors, reaffirming the significance of this observation.

The underlying mechanisms linking temperature with mortality in sepsis are multifaceted. Hypothermia can contribute to immune suppression, impaired thermoregulation, and disrupted coagulation pathways by causing platelet dysfunction and a mild decrease in platelet count or other steps in the coagulation cascade, thereby exacerbating mortality risk.^27^ Conversely, fever has been linked to improved survival due to enhanced bacterial clearance, optimized antibiotic efficacy, and controlled inflammatory responses.^11^ ^,^ ^28^ In contrast to prior studies in sepsis populations, our findings in the sepsis-induced coagulopathy (SIC) cohort did not indicate that an early peak temperature above 39.5°C was associated with worse outcomes.^11^ ^,^ ^29^ In fact, patients with temperatures in the 37.0–38.0°C range exhibited the lowest mortality, similar to those with temperatures above 39.0°C.

We found that normothermia (37.0–38.0°C, defined as a mild fever in some countries) was associated with reduced mortality compared to 36.0–37.0°C. This is consistent with previous findings. A multicenter RCT by Schortgen et al. reported external cooling to achieve normothermia (36.5–37.0°C) could reduce 14-day mortality compared to external heating to achieve fever control.^8^ However, a RCT by Young et al. did not find a significant difference in 90-day mortality between fever control and no fever control.^30^ The discrepancy may be explained by the different target temperature ranges. Overall, maintaining normothermia or mild fever appears to be beneficial based on current evidence.

Fever is common in sepsis patients. However, studies on the association between high fever (>39.0°C) and sepsis mortality remain controversial.^16^ ^,^ ^31^ Our study showed slightly increased mortality in high fever patients with SIC than in mild fever patients, but did not reach statistically significance Future research is warranted to clarify this relationship in SIC patients.

Moreover, our results demonstrated a nonlinear relationship between body temperature and both 28-day and 90-day all-cause mortality. Each Celsius degree increase in temperature correlated with an 18% reduction in 28-day mortality, underscoring the potential benefits of maintaining normothermia during SIC treatment. However, the relationship shifted beyond a temperature threshold of 38.0°C, where consistently increased temperatures correlated with gradual, albeit statistically insignificant, increases in mortality.

Notably, subgroup analysis revealed no significant interaction on 28-day mortality in most subgroups, suggesting that body temperature might independently influence the prognosis of SIC patients regardless of factors like age, gender, or other comorbidities. However, a significant interaction was observed in cerebrovascular disease patients, indicating that this specific subgroup may have unique thermoregulation characteristics warranting further investigation.^32^ The phenomenon that patients with sepsis complicated by cerebrovascular disease displayed different patterns of fever and outcome associations merits closer investigation. Several possible mechanisms may underpin this interaction. First, thermoregulation in cerebrovascular disease is impaired. Central thermoregulation involves complex neural circuitry like the preoptic anterior hypothalamus.^33^ Ischemic or hemorrhagic stroke can interrupt the involved pathways, compromising thermoregulatory capacity.^34^ This may contribute to altered fever responses in our subgroup of patients. Second, cerebral inflammation is exacerbated by fever. Animal studies showed hyperthermia can worsen ischemic injuries via increased neutrophil infiltration, blood-brain barrier dysfunction, edema formation and neuronal loss.^35^ ^,^ ^36^ Therefore, febrile responses in sepsis patients with preexisting cerebrovascular lesions may worsen secondary insults through neuroinflammation. Third, cerebral perfusion is impaired by fever. Fever escalates metabolic demands while septic shock reduces cerebral perfusion.^37^ ^–^ ^39^ The combined effects may create mismatches in oxygen supply and demand, setting the stage for ischemic damage. Strict fever control might help conserve neuronal viability.

Our study holds several notable strengths. By exclusively focusing on SIC patients, we address a crucial gap in the literature. Our comprehensive analysis bolsters the reliability and generalizability of our findings, while also uncovering novel correlations and their implications. The observed non-linear temperature-mortality relationship adds a new layer of understanding to SIC management, highlighting potential harm at both temperature extremes.

However, certain limitations should be acknowledged. Our study’s retrospective nature and reliance on data from a single center introduce potential biases and limit external validity. Inclusion of patients receiving antipyretics or temperature management could influence observed temperatures. Additionally, the use of the highest body temperature at ICU admission may not capture dynamic temperature changes. It is also important to note that our analysis was limited to 9,860 patients out of the initial cohort of 23,828 patients due to the lack of available SIC scores, which was our primary independent variable of interest. The exclusion of patients without SIC scores may have introduced potential selection bias, as these patients could have differed systematically from those included in the analysis. However, the large sample size of 9,860 patients with complete data still provides a robust basis for our findings. Future studies with more comprehensive data collection would be valuable to further validate and generalize our results.

## CONCLUSION

In conclusion, our study identifies an association that merits attention in the context of SIC patient management in ICU settings. We found that among SIC patients on their first day in the ICU, a body temperature from 37.0–38.0°C was associated with a significantly improved prognosis, marked by the lowest overall mortality risk, while the group with low body temperature (<36.0°C) exhibited the highest mortality risk. Additionally, a gradual but statistically non-significant increase in mortality risk was observed when body temperature exceeded 38.0°C. Future research on the impact of rigorous temperature management on SIC patient outcomes, and prospective validation of our association between temperature and sepsis mortality, is needed to provide a more comprehensive understanding of this complex relationship.

## Supplementary Information




## Data Availability

All data in the article can be obtained from MIMIC-IV database (https://mimic.physionet.org/).
