# Supplementary material for: The Nonlinear Relationship Between Temperature and Prognosis in Sepsis-induced Coagulopathy Patients: A Retrospective Cohort Study from MIMIC-IV Database
Source: West J Emerg Med. 2024 Aug 16;25(5):697–707. doi: 10.5811/westjem.18589 (PMC11418858; doi:10.5811/westjem.18589)
Supplement: Supplementary file 1 [file wjem-25-697-s001.docx]

| **Table S1** Missing number (%) of risk variables and outcome variables | | |
| --- | --- | --- |
| **Variables** | **Missing number** | **Missing percentiage(%)** |
| Age | 0 | 0 |
| Race | 0 | 0 |
| Gender | 0 | 0 |
| HR | 0 | 0 |
| RR | 3 | 0.030 |
| WBC | 2 | 0.020 |
| HGB | 4 | 0.041 |
| AG | 2 | 0.020 |
| CO_2_ | 2 | 0.020 |
| PLT | 0 | 0 |
| Glucose | 56 | 0.568 |
| BUN | 2 | 0.020 |
| Scr | 1 | 0.010 |
| INR | 0 | 0 |
| PT | 0 | 0 |
| PTT | 45 | 0.456 |
| Myocardial infarct | 0 | 0 |
| Congestive heart failure | 0 | 0 |
| Cerebrovascular disease | 0 | 0 |
| Chronic pulmonary disease | 0 | 0 |
| Diabetes | 0 | 0 |
| Renal disease | 0 | 0 |
| Charlson comorbidity index | 0 | 0 |
| SOFA score | 0 | 0 |
| SAPSII | 0 | 0 |
| RRT use | 0 | 0 |
| Ventilator use | 0 | 0 |
| Vasopressor use | 0 | 0 |
| Los hospital | 0 | 0 |
| Los ICU | 0 | 0 |
| 28-day mortality | 0 | 0 |
| 90-day mortality | 0 | 0 |
| Abbreviations: *HR*: heart rate, *RR*: respiratory rate, *WBC*: white blood cell, *HGB:* hemoglobin, *PLT*: platelet, *AG*: anion gap, *CO_2_*：bicarbonate，*BUN*: blood urea nitrogen, *Scr*: serum creatinine, *INR*: International Normalized Ratio, *PT*: prothrombin time, *PTT*: partial thromboplastin time, *SOFA*: sequential organ failure assessment, *SAPSII*: simplified acute physiology score II, *RRT*: renal replacement therapy, *LOS*: length of stay. | | |

| Table S2 Potential risk variables for the 28-day mortality | | |
| --- | --- | --- |
| **Variable** | **OR (95%CI)** | ***P-*value** |
| Temperature | 0.78 (0.73~0.83) | <0.001 |
| Age | 1.02 (1.02~1.02) | <0.001 |
| Race | 1.4 (1.27~1.55) | <0.001 |
| Gender | 1.32 (1.19~1.45) | <0.001 |
| HR | 1.01 (1.01~1.02) | <0.001 |
| RR | 1.04 (1.03~1.04) | <0.001 |
| HGB | 0.96 (0.94~0.98) | 0.001 |
| PLT | 1 (1~1) | 0.063 |
| WBC | 1.01 (1~1.01) | <0.001 |
| AG | 1.17 (1.16~1.19) | <0.001 |
| CO2 | 0.93 (0.92~0.94) | <0.001 |
| BUN | 1.02 (1.02~1.02) | <0.001 |
| Scr | 1.18 (1.15~1.21) | <0.001 |
| Glucose | 1 (1~1) | <0.001 |
| INR | 1.21 (1.17~1.24) | <0.001 |
| PT | 1.02 (1.02~1.02) | <0.001 |
| PTT | 1.01 (1.01~1.01) | <0.001 |
| Myocardial infarct | 1.19 (1.06~1.35) | 0.005 |
| Congestive heart failure | 1.41 (1.28~1.57) | <0.001 |
| Cerebrovascular disease | 1.67 (1.46~1.92) | <0.001 |
| Chronic pulmonary disease | 1.16 (1.04~1.29) | 0.01 |
| Diabetes | 0.95 (0.85~1.05) | 0.31 |
| Renal disease | 1.49 (1.33~1.66) | <0.001 |
| Charlson comorbidity index | 1.24 (1.22~1.26) | <0.001 |
| Sofa score | 1.15 (1.13~1.18) | <0.001 |
| Sapsii | 1.06 (1.06~1.06) | <0.001 |
| RRT | 2.19 (1.84~2.61) | <0.001 |
| Firstday ventilation | 1.11 (1.01~1.22) | 0.034 |
| Vasopressor use | 1.03 (0.93~1.13) | 0.618 |
| Abbreviations: *OR*: odds ratio, *CI*: confidence interval, *HR*: heart rate, *RR*: respiratory rate, *WBC*: white blood cell, *HGB:* hemoglobin, *PLT*: platelet, *AG*: anion gap, *CO_2_*：bicarbonate，*BUN*: blood urea nitrogen, *Scr*: serum creatinine, *INR*: International Normalized Ratio, *PT*: prothrombin time, *PTT*: partial thromboplastin time, *SOFA*: sequential organ failure assessment, *SAPSII*: simplified acute physiology score II, *RRT*: renal replacement therapy. | | |

| Table S3 Potential risk variables for the 90-day mortality | | |
| --- | --- | --- |
| **Variable** | **OR (95%CI)** | ***P*-value** |
| Temperature | 0.78 (0.74~0.83) | <0.001 |
| Age | 1.02 (1.02~1.02) | <0.001 |
| Race | 1.29 (1.17~1.41) | <0.001 |
| Gender | 1.29 (1.18~1.41) | <0.001 |
| HR | 1.01 (1.01~1.01) | <0.001 |
| RR | 1.03 (1.03~1.04) | <0.001 |
| HGB | 0.95 (0.93~0.97) | <0.001 |
| PLT | 1 (1~1) | <0.001 |
| WBC | 1.01 (1~1.01) | <0.001 |
| AG | 1.17 (1.15~1.18) | <0.001 |
| CO2 | 0.93 (0.92~0.94) | <0.001 |
| BUN | 1.02 (1.02~1.02) | <0.001 |
| Scr | 1.19 (1.16~1.22) | <0.001 |
| Glucose | 1 (1~1) | <0.001 |
| INR | 1.22 (1.18~1.26) | <0.001 |
| PT | 1.02 (1.02~1.02) | <0.001 |
| PTT | 1.01 (1.01~1.01) | <0.001 |
| Myocardial infarct | 1.18 (1.06~1.32) | 0.004 |
| Congestive heart failure | 1.63 (1.48~1.79) | <0.001 |
| Cerebrovascular disease | 1.67 (1.47~1.9) | <0.001 |
| Chronic pulmonary disease | 1.18 (1.07~1.31) | 0.001 |
| Diabetes | 1.01 (0.92~1.12) | 0.778 |
| Renal disease | 1.58 (1.42~1.75) | <0.001 |
| Charlson comorbidity index | 1.29 (1.27~1.31) | <0.001 |
| Sofa score | 1.14 (1.12~1.16) | <0.001 |
| Sapsii | 1.06 (1.06~1.06) | <0.001 |
| RRT | 2.23 (1.89~2.63) | <0.001 |
| Firstday ventilation | 0.97 (0.89~1.06) | 0.453 |
| Vasopressor use | 0.91 (0.83~0.99) | 0.031 |
| Abbreviations: *OR*: odds ratio, *CI*: confidence interval, *HR*: heart rate, *RR*: respiratory rate, *WBC*: white blood cell, *HGB:* hemoglobin, *PLT*: platelet, *AG*: anion gap, *CO_2_*：bicarbonate，*BUN*: blood urea nitrogen, *Scr*: serum creatinine, *INR*: International Normalized Ratio, *PT*: prothrombin time, *PTT*: partial thromboplastin time, *SOFA*: sequential organ failure assessment, *SAPSII*: simplified acute physiology score II, *RRT*: renal replacement therapy. | | |


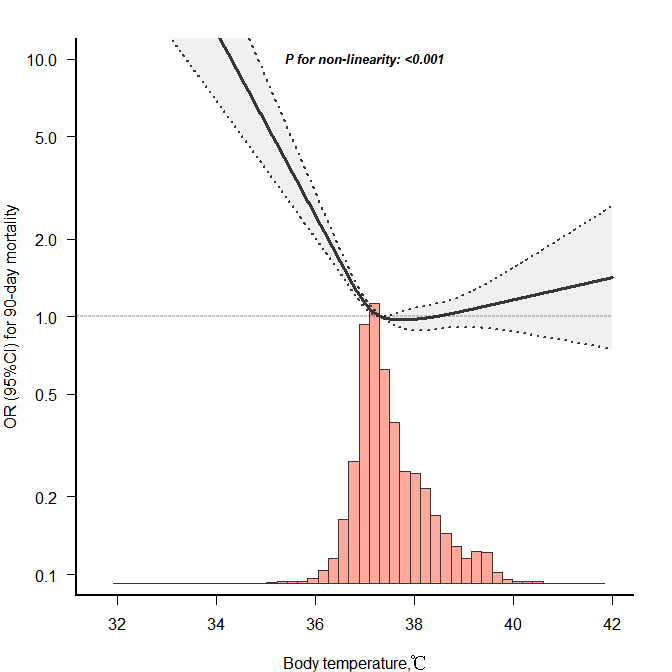


Figure S1 Smooth curve fitting for temperature and 90-day mortality in patients with sepsis-induced coagulopathy.


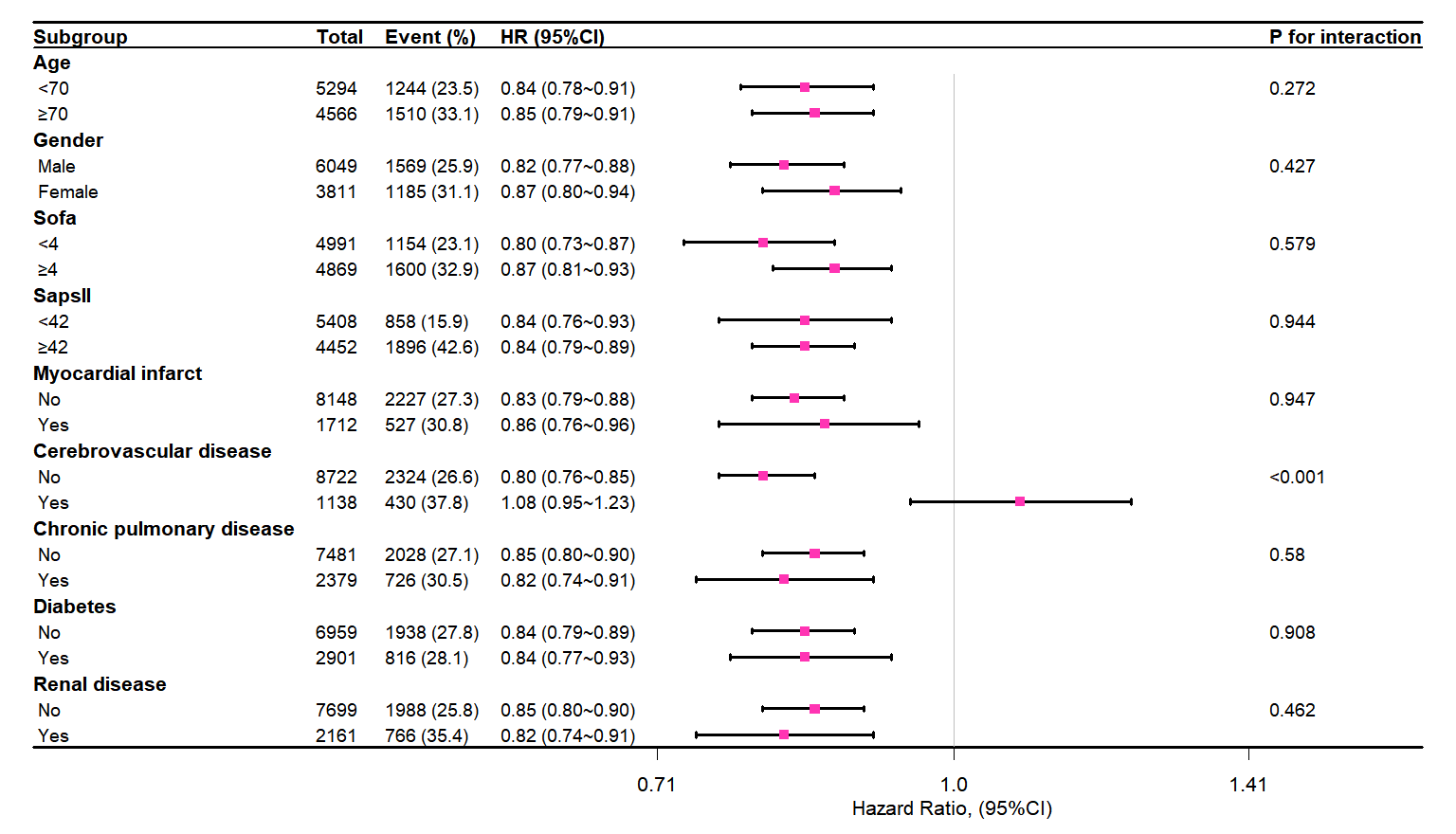


Figure S2 Smooth curve fitting for temperature and 90-day mortality in patients with sepsis-induced coagulopathy.


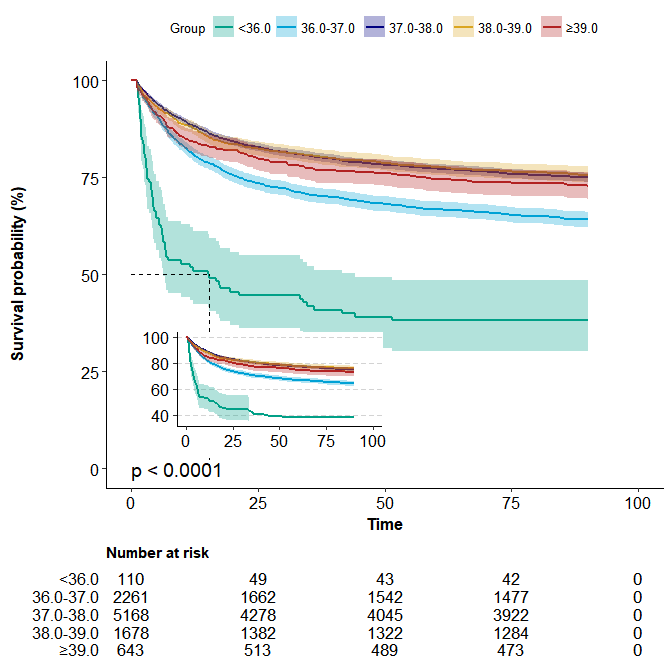


Figure S3 Kaplan–Meier curve of 90-day mortality for patients with sepsis-induced coagulopathy.
