# Supplementary material for: The Nonlinear Relationship Between Temperature and Prognosis in Sepsis-induced Coagulopathy Patients: A Retrospective Cohort Study from MIMIC-IV Database
Source: West J Emerg Med. 2024 Aug 16;25(5):697–707. doi: 10.5811/westjem.18589 (PMC11418858; doi:10.5811/westjem.18589)
Supplement: Supplementary file 2 [file wjem-25-697-s002.doc]

| Table S4 Results of multivariate regression analysis between temperature and outcomes. | | | | | | | | | | | |
| --- | --- | --- | --- | --- | --- | --- | --- | --- | --- | --- | --- |
| **Variable** | **Model I** | |  | **Model II** | |  | **Model III** | |  | **Model IV** | |
|  | **HR (95% CI)** | ***p***-value |  | **HR (95% CI)** | ***p***-value |  | **HR (95% CI)** | ***p***-value |  | **HR (95% CI)** | ***p***-value |
| **Primary outcomes** |  |  |  |  |  |  |  |  |  |  |  |
| 28-day mortality^a^ |  |  |  |  |  |  |  |  |  |  |  |
| Temperature | 0.78 (0.74~0.83) | <0.001 |  | 0.73 (0.69~0.78) | <0.001 |  | 0.82 (0.78~0.87) | <0.001 |  | 0.82 (0.78~0.87) | 0.001 |
| Body temperature |  |  |  |  |  |  |  |  |  |  |  |
| <36.0 | 2.82 (2.17~3.66) | <0.001 |  | 3.32 (2.55~4.33) | <0.001 |  | 2.79 (2.13~3.65) | <0.001 |  | 2.6 (1.99~3.42) | <0.001 |
| 36.0-37.0 | 1(Ref) |  |  | 1(Ref) |  |  | 1(Ref) |  |  | 1(Ref) |  |
| 37.0-38.0 | 0.62 (0.56~0.69) | <0.001 |  | 0.62 (0.56~0.69) | <0.001 |  | 0.7 (0.63~0.78) | <0.001 |  | 0.7 (0.63~0.78) | <0.001 |
| 38.0~-39.0 | 0.63 (0.55~0.72) | <0.001 |  | 0.59 (0.51~0.68) | <0.001 |  | 0.75 (0.65~0.86) | <0.001 |  | 0.76 (0.66~0.88) | 0.001 |
| ≥39.0 | 0.75 (0.62~0.9) | 0.002 |  | 0.61 (0.5~0.74) | <0.001 |  | 0.74 (0.61~0.91) | 0.003 |  | 0.72 (0.59~0.87) | 0.001 |
| Trend |  | <0.001 |  |  | <0.001 |  |  | <0.001 |  |  | <0.001 |
| **Secondary outcomes** |  |  |  |  |  |  |  |  |  |  |  |
| 90-day motality^a^ |  |  |  |  |  |  |  |  |  |  |  |
| Temperature | 0.79 (0.75~0.83) | <0.001 |  | 0.75 (0.71~0.79) | <0.001 |  | 0.83 (0.79~0.88) | <0.001 |  | 0.84 (0.8~0.89) | <0.001 |
| Body temperature |  |  |  |  |  |  |  |  |  |  |  |
| <36.0 | 2.52 (1.97~3.23) | <0.001 |  | 3.01 (2.35~3.86) | <0.001 |  | 2.49 (1.94~3.21) | <0.001 |  | 2.48 (1.92~3.2) | <0.001 |
| 36.0-37.0 | 1(Ref) |  |  | 1(Ref) |  |  | 1(Ref) |  |  | 1(Ref) |  |
| 37.0-38.0 | 0.64 (0.59~0.7) | <0.001 |  | 0.65 (0.59~0.71) | <0.001 |  | 0.73 (0.67~0.8) | <0.001 |  | 0.73 (0.67~0.8) | <0.001 |
| 38.0-39.0 | 0.63 (0.56~0.71) | <0.001 |  | 0.6 (0.53~0.68) | <0.001 |  | 0.75 (0.66~0.85) | <0.001 |  | 0.79 (0.69~0.89) | <0.001 |
| ≥39.0 | 0.72 (0.61~0.85) | <0.001 |  | 0.61 (0.52~0.73) | <0.001 |  | 0.74 (0.63~0.88) | 0.001 |  | 0.73 (0.62~0.87) | 0.001 |
| Trend |  | <0.001 |  |  | <0.001 |  |  | <0.001 |  |  | <0.001 |
| LOS ICU^b^ | 0.77^＊^  (0.61~0.92) | <0.001 |  | 0.39^＊^ (0.23~0.55) | <0.001 |  | 0.57^＊^ (0.42~0.73) | <0.001 |  | 0.4^＊^ (0.24~0.55) | <0.001 |
| LOS hospital^b^ | 1.33^＊^  (1.01~1.66) | <0.001 |  | 0.46^＊^  (0.12~0.8) | 0.008 |  | 0.84^＊^ (0.5~1.18) | <0.001 |  | 0.82^＊^ (0.48~1.15) | <0.001 |
| *Notes:*  Model I adjusted for nothing;  Model II adjusted for gender, age, race, HR, RR;  Model III adjusted for Model II plus hemoglobin, platelets, INR, wbc, anion gap, bicarbonate, bun, creatinine, glucose, PT, PTT.  Model IV adjusted for Model III plus myocardial infarction, congestive heart failure, cerebrovascular disease, chronic pulmonary disease, renal disease, charlson comorbidity index, SOFA score, sapsII, RRT, first day ventilation use, vasopressor use.  ^a^Logistic regression analysis  ^b^Linear regression analysis  ^＊^Regression coefficient (*β)*  Abbreviations: *HR*, hazard ratio; *CI*, confidence interval; *LOS:*length of stay. | | | | | | | | | | | |
